# Fecal Bacteria Implicated in Biofilm Production Are Enriched and Associate to Gastrointestinal Symptoms in Patients With APECED – A Pilot Study

**DOI:** 10.3389/fimmu.2021.668219

**Published:** 2021-07-22

**Authors:** Iivo Hetemäki, Ching Jian, Saila Laakso, Outi Mäkitie, Anne-Maria Pajari, Willem M. de Vos, T. Petteri Arstila, Anne Salonen

**Affiliations:** ^1^ Translational Immunology Research Program, University of Helsinki and Helsinki University Hospital, Helsinki, Finland; ^2^ Human Microbiome Research Program, Faculty of Medicine, University of Helsinki, Helsinki, Finland; ^3^ Institute of Genetics, Folkhälsan Research Center, Helsinki, Finland; ^4^ Children’s Hospital, University of Helsinki and Helsinki University Hospital, Helsinki, Finland; ^5^ Clinical and Molecular Metabolism Research Program, Faculty of Medicine, University of Helsinki, Helsinki, Finland; ^6^ Department of Molecular Medicine, Karolinska Institutet, and Clinical Genetics, Karolinska University Hospital, Stockholm, Sweden; ^7^ Department of Food and Nutrition, University of Helsinki, Helsinki, Finland; ^8^ Laboratory of Microbiology, Wageningen University, Wageningen, Netherlands

**Keywords:** dysbiosis, gut microbiota, immune dysregulation, lps, autoantibodies, atopobium, faecalibacterium, autoimmunity

## Abstract

**Backgrounds and Aims:**

APECED is a rare autoimmune disease caused by mutations in the Autoimmune Regulator gene. A significant proportion of patients also have gastrointestinal symptoms, including malabsorption, chronic diarrhea, and obstipation. The pathological background of the gastrointestinal symptoms remains incompletely understood and involves multiple factors, with autoimmunity being the most common underlying cause. Patients with APECED have increased immune responses against gut commensals. Our objective was to evaluate whether the intestinal microbiota composition, predicted functions or fungal abundance differ between Finnish patients with APECED and healthy controls, and whether these associate to the patients’ clinical phenotype and gastrointestinal symptoms.

**Methods:**

DNA was isolated from fecal samples from 15 patients with APECED (median age 46.4 years) together with 15 samples from body mass index matched healthy controls. DNA samples were subjected to analysis of the gut microbiota using 16S rRNA gene amplicon sequencing, imputed metagenomics using the PICRUSt2 algorithm, and quantitative PCR for fungi. Extensive correlations of the microbiota with patient characteristics were determined.

**Results:**

Analysis of gut microbiota indicated that both alpha- and beta-diversity were altered in patients with APECED compared to healthy controls. The fraction of *Faecalibacterium* was reduced in patients with APECED while that of *Atopobium* spp. and several gram-negative genera previously implicated in biofilm formation, e.g. *Veillonella, Prevotella, Megasphaera* and *Heamophilus*, were increased in parallel to lipopolysaccharide (LPS) synthesis in imputed metagenomics. The differences in gut microbiota were linked to patient characteristics, especially the presence of anti-*Saccharomyces cerevisiae* antibodies (ASCA) and severity of gastrointestinal symptoms.

**Conclusions:**

Gut microbiota of patients with APECED is altered and enriched with predominantly gram-negative bacterial taxa that may promote biofilm formation and lead to increased exposure to LPS in the patients. The most pronounced alterations in the microbiota were associated with more severe gastrointestinal symptoms.

## Highlights

APECED is a rare autoimmune disease caused by mutations in the Autoimmune Regulator gene. A significant proportion of patients have gastrointestinal symptoms, including malabsorption, chronic diarrhea, and obstipation that lead to decrease in the quality of life. The pathological background of the gastrointestinal symptoms remains incompletely understood. We have previously found that patients with APECED have increased immune responses against gut commensals, but previous smaller studies have found only small alterations in the microbiota of the gut of the patients. Our objective was to evaluate whether the intestinal microbiota composition differs between patients with APECED (N=15) and healthy controls, and whether these are associated with the patients’ clinical phenotype and gastrointestinal symptoms. We found both alpha- and beta-diversity to be altered in patients with APECED compared to healthy controls. Several gram-negative genera previously implicated in biofilm formation were increased in patients, in parallel with lipopolysaccharide (LPS) synthesis in imputed metagenomics. Interestingly, the most pronounced changes in the microbiota were associated with more severe gastrointestinal symptoms in patients with APECED, suggesting that gut microbiota is a factor to consider when contemplating therapy.

## Introduction

APECED (autoimmune polyendocrinopathy-candidiasis-ectodermal dystrophy, OMIM #240300) is a rare autoimmune disease caused by autosomal recessive mutations in the gene encoding the Autoimmune Regulator (AIRE) (1). AIRE is expressed in medullary thymic epithelial cells where it regulates the expression of tissue-restricted antigens, most likely contributing to thymic negative selection. The lack of a fully functioning AIRE leads to autoimmunity against multiple endocrine organs, resulting in hormonal deficiencies of which hypoparathyroidism and primary adrenal insufficiency are the most common manifestations (1). Practically all patients with APECED have neutralizing anti-cytokine antibodies some of which have been linked susceptibility to *Candida albicans* infections (2).

A significant proportion of patients with APECED present with gastrointestinal (GI) symptoms, such as malabsorption, chronic diarrhea, obstipation, and gastritis (3). The pathological background of the GI symptoms remains incompletely understood, but they have been attributed to multiple factors with autoimmunity being the most common underlying cause. About half of patients with APECED have autoimmunity against gut neuroendocrine cells associated with antibodies against tryptophan hydroxylase (TPH) (4). The loss of neuroendocrine cells leads to decreased serum serotonin levels, which in turn is associated with symptoms of obstipation (5). In addition, around a quarter of the patients exhibit autoimmunity against defensins, culminating in a loss of Paneth cells (6). Anti-defensin antibodies are linked to gut microbiota dysbiosis in Aire -/- mice and diarrhea in humans (6). Autoimmune hepatitis, gastritis, and exocrine pancreatic malfunction also affect patients’ GI health.

We have previously detected anti-*Saccharomyces cerevisiae* antibodies (ASCA) and other anti-commensal antibodies in patients with APECED, in a pattern reminiscent of the one seen in Crohn’s disease (7). It has been suggested that an overt inflammatory response against normal gut microbiota plays a vital role in the pathogenesis of Crohn’s disease, and a deviating gut microbiota, characterized by a decrease of butyrate producers and an increase of pro-inflammatory Proteobacteria, has been observed in a significant number of patients with Crohn’s disease (8–11). Previous reports on small numbers of patients (10 and 11 patients) offer some indication that deviations in the gut microbiota might also be a feature of APECED, but the differences between patients and controls have been modest (5, 6). Here we present a characterization of the gut microbiota in, to our knowledge, the largest cohort of APECED patients described so far, showing clear aberrations of the gut microbiota. These changes are similar to the ones observed in Crohn’s disease, more pronounced in patients with anti-*Saccharomyces cerevisiae antibodies*, and are associated with chronic diarrhea.

## Materials and Methods

We invited all patients who have taken part in previous large Finnish clinical studies on APECED (1) to participate in the current study. Additional patients were recruited from all Finnish university hospitals and central hospitals by contacting the respective endocrine units. Out of the 91 Finnish patients enrolled in previous studies (6), 61 were alive at the time of recruitment. Altogether 37 (68%) adult patients consented and were included in the study during the years of 2015-2016 (12). Fifteen patients (seven females), in the age range of 19 to 70 years (mean, 45.9 years) with a body mass index (BMI) range of 17.7 to 40.7 kg/m^2^ (mean, 23.2 kg/m^2^) were willing to donate frozen fecal sample and form the study patient group. Fifteen control fecal samples, from healthy individuals (ten females) aged between 20 to 67 years (mean, 34.9 years) with a BMI between 18.1 to 33.0 (mean, 23.9), were selected from an inhouse database to be used as controls. The controls were selected to match patients as closely as possible in relation to age, BMI and sex. Exclusion criteria in the control group were medication for hypercholesterolemia or hypertension, regular or recent (within the past three months) use of antibiotics, extreme sport, smoking, pregnancy, or lactation. Sample processing methodology from sampling to DNA extraction and sequencing was identical between the patient and control samples.

The Ethics committee of the Helsinki University Hospital approved the study protocol, and subjects gave their signed informed consent prior to enrollment. The study was performed according to the principles of the Declaration of Helsinki.

### Clinical Data

Clinical details for APECED patients were collected from medical records, through a questionnaire and patient interviews, which included questions on medical history, prior infections, medications, and other relevant parameters. All patients were clinically examined by a medical doctor (S.L.). The grading of the severity of GI symptoms experienced by the patients was done based on discussion and a unanimous decision by two physicians (I.H. and S.L.). The severity of diarrhea was classified according to the degree of symptoms experienced: 1) no symptoms, 2) intermittent diarrhea, episodes of diarrhea separated by obstipation or symptom-free periods, 3) chronic difficult diarrhea.

### Sample Collection and Processing of Fecal Samples

Fecal samples for the patients and controls were collected at home and immediately stored at -20°C. They were transported to a study center within 1 week. An uninterrupted frozen cold chain was ensured in the provision and handling of the fecal samples. Bacterial DNA was extracted from ca. 250 mg of fecal matter using the Repeated Bead Beating (RBB) method (13) with the following modifications for automated DNA purification: 340 μl and 145 μl of lysis buffer was added to first and second round of bead beating, respectively. 200 µl of the clarified supernatant collected from the two bead beating rounds was used for DNA extraction with the Ambion Magmax™ -96 DNA Multi-Sample Kit (Thermo Fisher Scientific, USA) using the KingFisherTM Flex automated purification system (Thermo Fisher Scientific, USA). DNA was quantified using Quanti-iT™ Pico Green dsDNA Assay (Invitrogen, San Diego, CA, USA).

Blood samples were collected in the morning between 7 and 10 am after an 8- to 12-hour fast before taking morning medications. Sera were isolated with a standard protocol and stored at -80°C until analyses.

### Quantification of Anti-*Saccharomyces cerevisiae* Antibodies

The ASCA were quantified from patient sera with the Anti-*Saccharomyces cerevisiae* Antibodies IgG test kit (Bio-Rad, USA) as instructed by the manufacturer. Values over 15 U/mL were considered positive.

### Anti-Tryptophan Hydroxylase Antibodies

Immunoprecipitation of radiolabeled antigens was used to screen sera for autoantibodies against tryptophan hydroxylase-1. Human complementary DNA from the antigen in expression vector was used to perform the assay. *In vitro* transcription and translation were performed in the presence of ^35^S-methionine, according to the manufacturer’s protocol (Promega TNT Systems). Immunoprecipitation was performed in 96-well plates overnight at 4°C at 300 rpm with serum samples (2.5 µl) and 30,000 cpm of radiolabeled protein. A positive control (positive patient serum to each antigen) and a negative control, 4% bovine serum albumin, were included in each plate. All samples were analyzed in duplicates. The immune reaction was transferred to filter plates (Millipore) and immune complexes were captured to Protein A sepharose (nProtein A Sepharose 4 fast flow, GE Healthcare) during a 45 min incubation at 4°C at 300 rpm. After ten washing steps with wash buffer (150mM NaCl, 20 mM TrisHCl pH 8, 0.15% Tween 20, and 0.1% BSA), plates were dried and scintillation fluid (Optiphase, HiSafe 3, PerkinElmer) was added. Radioactivity was then measured in a beta counter (1450 Microbeta Trilux, Wallac). Autoantibody index values were calculated according to the following: (sample value – negative control value)/(positive control value – negative control value) x 100.

### Quantitative PCR for Fungi

For qPCR targeting fungal ITS1-region, 5 ng of DNA was mixed with 2 μM of ITS1F and ITS2 primers (14) and 2X Power SYBR green PCR Master Mix (Thermo Fisher, USA) in a total volume of 20 μl. The PCR reaction was performed in the C1000 Touch Thermal Cycler (Bio-Rad, USA) as follows: 95°C for 15 min, followed by 40 cycles of 95°C 15 sec, 55°C 30 sec and 72°C 30 sec and ending in 95°C 1 min and 60°C 1 min. Results were analyzed using the comparative Ct method (15).

### Microbiota Analysis

Library preparation and Illumina MiSeq sequencing of the hypervariable V3-V4 regions of the 16S rRNA gene and sequencing data preprocessing for the patient and control samples as well as negative controls were performed as previously described (16).

### Sequencing Data Preprocessing Analysis and Statistics

The pre-processing of the sequencing reads, their taxonomic annotation, and statistical analysis were performed using R package *mare* (Microbiota Analysis in R Easily) (17) as previously described (16, 18). Samples from cases and controls were sequenced in two different MiSeq runs with non-template negative controls. The lowest number of reads per sample was 28175 and the mean number of reads was 72610 for patients and 73661 for controls. The three negative control samples in the run with APECED samples contained 139, 259, and 728 reads assigned to two uncultured lactobacilli. As the reads assigned to *Lactobacillus* in the APECED samples were also mostly assigned to the two uncultured lactobacilli in negative controls, we excluded *Lactobacillus* from all data analyses. To account for the varying sequencing depth, the number of reads per sample was used as an offset in all statistical models. The β-diversity was estimated using Bray-Curtis dissimilarity as the distance measure and the contribution of different variables to microbiota variation was calculated using permutational ANOVA (PERMANOVA) *adonis* function in *vegan* (19) on genus, family and phylum levels. The BMI or gender were not associated with microbiota variation (permutational ANOVA 4% and 3%, respectively, FDR-*P* > 0.3 for both) and were not used as confounding factors. Also, age did not have a statistically significant effect on permutational ANOVA, but it correlated with the relative abundance of some of bacteria of interest in patients (*Faecalibacterium, Megasphaera* and *Prevotella*) so it was used as a confounder in all models. The differences in the microbiota between patients and controls or between patients with different clinical characteristics were analyzed using generalized linear models with negative binomial distribution implemented in the *mare* package for prevalent bacteria i.e. bacterial genera detected in > 60% of the samples. P-values were adjusted by the Benjamini-Hochberg method for multiple testing and reported as FDR*-P*. All significant findings from differential abundance testing underwent visual inspection when applicable to eliminate statistically significant findings driven by few extreme values. Only statistically significant and visually validated results are reported. In the univariate data, a statistical difference was evaluated using the Independent-Samples Mann-Whitney U test in SPSS 25 (IBM). For all tests, *P*-values < 0.05 were considered as statistically significant.

### Imputed Metagenomic Analysis

Bacterial metagenome content was predicted from the 16S rRNA gene-based microbial compositions. Functional inferences were made from the Kyoto Encyclopedia of Gene and Genomes (KEGG) catalog (20) using PICRUSt2 (21). The “*DESeq*” function in DESeq2 (22) was used to test for differentially abundant KEGG pathways between the groups.

### Random Forest Classification

Random forest model was used to evaluate the predictive performance of all identified genera (N = 65) or predicted functions (N = 222) based on leave-one-out cross-validation. The random forest analysis was performed using the default settings of the “randomForest” function implemented in the *randomForest* R package. We then used the receiver operating characteristic (ROC) curve and calculated the area under the ROC curve (AUC), implemented in the *pROC* package, as the measure of performance. The importance of input features was evaluated by mean decrease Gini values.

## Results

### Wide Spectrum of Intestinal Manifestations in the Patients With APECED

The microbiome samples of altogether 15 patients (7 females) with APECED were investigated. The patients were examined at the median age of 46.4 years (range, 19.3 – 70.1). Characteristics of the patients are presented in **Table 1** in comparison to 22 patients with APECED from whom we were unable to acquire stool samples. The patient characteristics differ little between the two groups suggesting that the sample of patients participating in the study represents well the overall APECED population.

**Table 1:** Clinical characteristics of the 15 adult patients with APECED included in the present study.

Characteristic	Patient with microbiome analyses	Patients without microbiome analyses	*P*-value
N (%) or median (range)	N = 15	N = 22	
Female	7 (47%)	15 (68%)	0.19
Age (year)	46.4 (19.3 – 70.1)	39.5 (21.9 – 62.7)	0.26
Height SDS	-1.5 (-2.9 - +1.3)	-1.2 (-2.6 - +1.4)	0.13
BMI (kg/m^2^)	22.7 (17.7 – 40.7)	22.3 (15.0 – 36.6)	0.87
AIRE genotype			
c.769C>T / c.769C>T	12 (80%)	18 (82%)	
c.769C>T / c.967_979del13	2 (13%)	2 (9%)	
c.769C>T / other	1 (7%)	2 (9%)	
Diarrhea	6 (40%) / 6 (40%) / 3 (20%)	9 (41 %) / 10 (45%) / 3 (14%)	0.82
(No / episodic / chronic)			
Obstipation	4 (27%)	10 (46%)	0.31
Failure of exocrine pancreas	0	4 (18)	0.13
Anti-fungal medication	11 (73%) / 3 (20%) / 1 (7%)	15 (68%) / 3 (14%) / 4 (18%)	0.65
(No / preventive / treatment dose)			
Probiotics in use	4 (27%)	1 (5%)	0.14

Their clinical and genetic features did not differ from the 22 patients with APECED for whom no data on stool microbiome was available.

All patients harbored at least one copy of the Finnish founder mutation c.769C>T, p.(Arg257Ter) in the *AIRE* gene (NM_000383.4). Altogether 13/15 of patients had primary adrenal insufficiency, and 12/15 had hypoparathyroidism. The patients had median of 7 (range 4-11) disease components of APECED. Additional symptoms, each present in one individual, included high blood pressure, asthma, depression, migraine, APC resistance, hypercholesterolemia and mild hypertrophic cardiomyopathy. All 15 patients had experienced mucocutaneus candidiasis. One patient was using anti-fungal medication with treatment dose and three patients with prophylaxis dose. Either periodic or chronic diarrhea was evident in 60% of the patients. None of the patients had used antibiotics within 1 month prior to the sampling. Four patients were using probiotics at the time of sampling, but these patients did not significantly differ from other patients in their microbial composition (data not shown). None of the controls had used antibiotics within 3 months prior to the sampling and none of them used probiotics.

### Microbial Composition of Patients With APECED Is Altered Compared to Healthy Controls

We performed 16S rRNA gene amplicon sequencing to investigate the microbial composition of the stool samples. After processing we had on average 73135 (28175-164233) high quality reads representing overall of 395 operational taxonomic units (OTUs) distributed to 78 bacterial genera, 33 families, 19 orders, 13 classes, and 5 phyla. The microbiota richness ranged from 94 to 220 (mean, 168 *vs.* 152 in patients and controls, respectively; not significant; Mann-Whitney U test). Alpha diversity was significantly higher in patients compared to healthy controls [mean 13.0 (range, 4.2-18.4) *vs.* 6.8 (4.3-10.5); *P* < 0.001; Mann Whitney U test].

**Figure 1:**
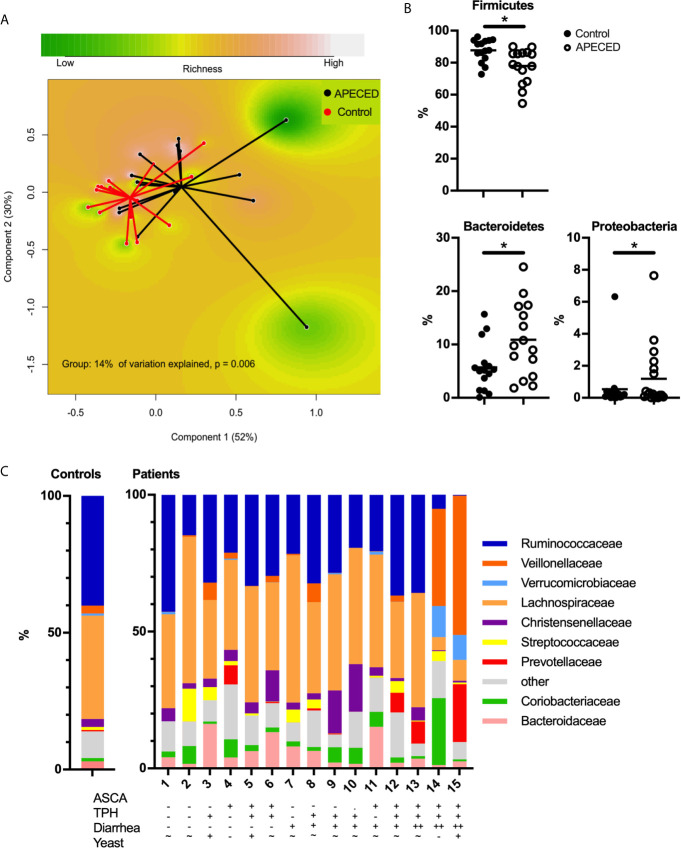
**(A)** Principal coordinate analysis (PCoA) based on Bray–Curtis distances using the phylum-level data showing differences in the gut microbiota compositions between patients with APECED (black) and healthy controls (red). **(B)** The relative abundances of Firmicutes, Bacteroidetes and Proteobacteria in patients with APECED (open circles) compared to healthy controls (black circles). * indicates FDR-*P*-value < 0.05 **(C)** Composition of the gut microbiota at the family level of patients with APECED and healthy controls. For readability, the composition of the controls is shown as mean relative abundance. Anti-*Saccharomyces cerevisiae* antibodies (ASCA), antibodies against tryptophan hydroxylase (TPH), gastrointestinal symptoms and results of stool yeast analysis are shown for each patient. – negative/low, + positive, ++ markedly positive i.e. severe symptoms, ~ average.

We compared the difference between patients and controls in the overall gut microbiota using PERMANOVA based on Bray-Curtis dissimilarity. Patients differed from controls already on the phylum level (14% of variation explained, *P* = 0.006, **Figure 1A**). Bacteroidetes and Proteobacteria were expanded in patients’ gut microbiota, while a lower proportion of Firmicutes was found (**Figure 1B**) compared to the controls. On the family level, PERMANOVA indicated a 10% variation in the gut microbiota between the patients and controls (*P* = 0.003). Two patients distinctly differed from others with markedly expanded relative abundance of *Veillonellaceae* and diminished *Ruminococcaceae* (**Figure 1C**). Interestingly, these subjects were two of the three patients reporting the most severe diarrhea.

**Figure 2:**
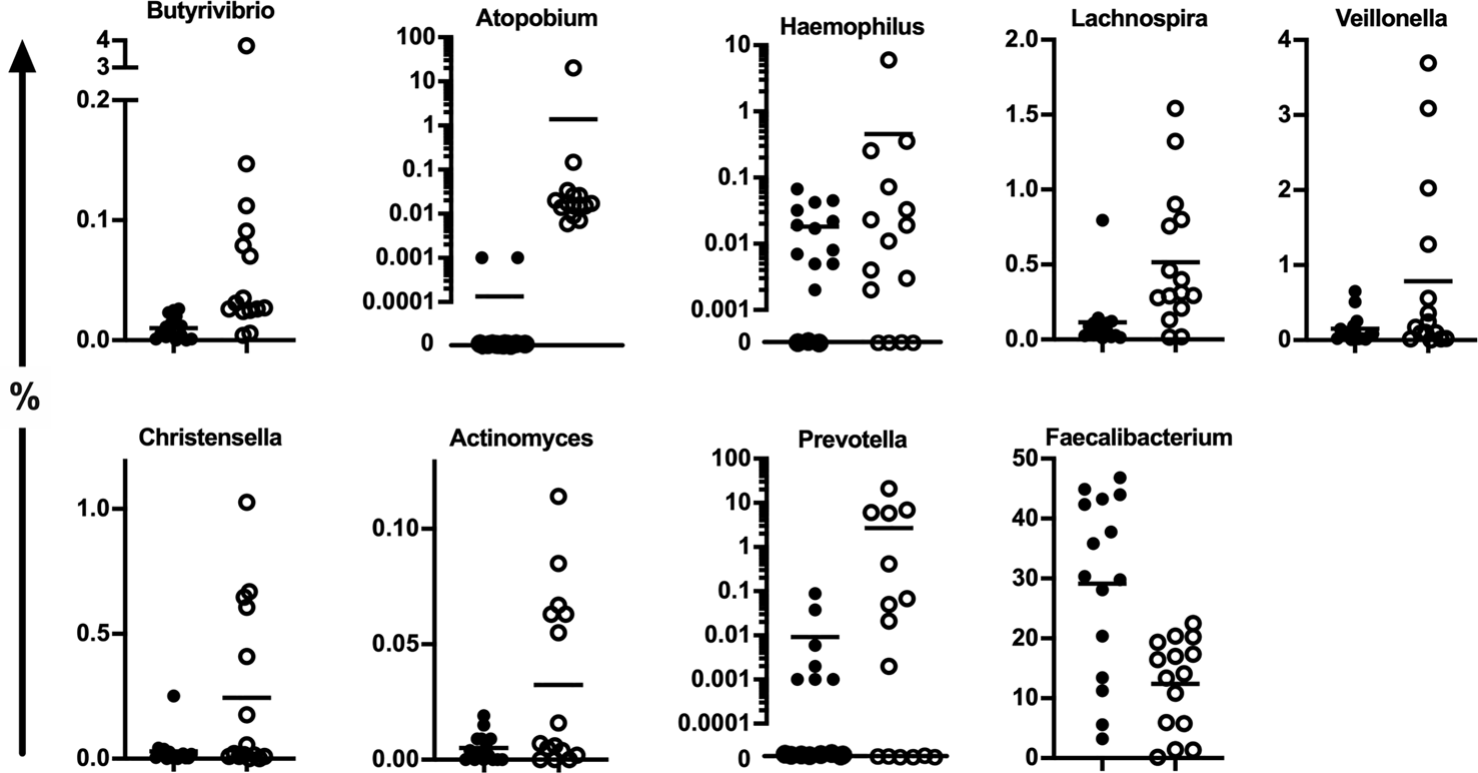
The relative abundance of all the bacterial genera that significantly altered in APECED patients’ stool (open circles) compared to healthy controls (black circles) based on the age-adjusted negative binomial models. The results of statistical analysis are summarized in **Table 2**.

Beta-diversity between the patients and controls was significantly different also on genus level (10% variation in PERMANOVA, *P* = 0.001). In comparison to healthy controls, the proportion of *Faecalibacterium* was significantly reduced in patients [fold change (fc) = 0.45, FDR-*P* = 0.05, **Table 2**, **Figure 2**]. Multiple genera were significantly overrepresented in the patients. The largest 15-20 fold increase was found in the fraction of *Butyrivibrio*, *Atopobium*, and *Haemophilus* (fc = 15.1, FDR-*P* = 7x10^-5^; fc = 18.8, FDR-*P* = 7x10^-5^; fc = 17.3, FDR-*P* = 0.0003 respectively, **Table 2** and **Figure 2**). *Atopobium* was almost undetectable in the majority of the controls while it was observed in all patients accounting up to 20.3% of the total microbiota in one patient. The relative abundance of other gram-negative bacteria, *Veillonella* and *Prevotella*, was significantly increased in patients (fc = 5.4, FDR-*P* = 0.02; fc = 2.0, FDR-*P* = 0.04; respectively, **Table 2** and **Figure 2**). Of interest, the reads derived from *Megasphaera* spp. within the *Veillonnellaceae family* accounted for up to 37.1% and 0.8% of reads in two patients, respectively, but was otherwise virtually undetectable in the majority of patients and controls (**Figure 2**). All genera with relative abundances that are significantly different between the patients and controls are summarized in **Table 2**.

**Table 2:** Bacterial genera that differed significantly between patients with APECED and controls.

Phylum	Class	Order	Family	Genus	Mean (%)	Fold Change	Log2 FC	FDR-*P*
Firmicutes	Clostridia	Clostridiales	*Lachnospiraceae*	*Butyrivibrio*	0.30	15.14	3.92	1.1E-05
Actinobacteria	Coriobacteriia	Coriobacteriales	*Coriobacteriaceae*	*Atopobium*	1.38	18.77	4.23	7.2E-05
Proteobacteria	Gammaproteobacteria	Pasteurellales	*Pasteurellaceae*	*Haemophilus*	0.45	17.27	4.11	0.0003
Firmicutes	Clostridia	Clostridiales	*Lachnospiraceae*	*Lachnospira*	0.52	4.41	2.14	0.0003
Firmicutes	Negativicutes	Selenomonadales	*Veillonellaceae*	*Veillonella*	0.78	5.40	2.43	0.02
Firmicutes	Clostridia	Clostridiales	*Christensenellaceae*	*Christensenella*	0.24	7.05	2.82	0.02
Actinobacteria	Actinobacteria	Actinomycetales	*Actinomycetaceae*	*Actinomyces*	0.03	5.08	2.34	0.03
Bacteroidetes	Bacteroidia	Bacteroidales	*Prevotellaceae*	*Prevotella*	2.70	2.00	1.00	0.04
Firmicutes	Clostridia	Clostridiales	*Ruminococcaceae*	*Faecalibacterium*	12.41	0.45	-1.16	0.05
Bacteroidetes	Bacteroidia	Bacteroidales	*Porphyromonadaceae*	*Parabacteroides*	0.36	2.90	1.53	0.07
Actinobacteria	Coriobacteriia	Coriobacteriales	*Coriobacteriaceae*	*Collinsella*	2.24	3.21	1.68	0.07
Bacteroidetes	Bacteroidia	Bacteroidales	*Bacteroidaceae*	*Bacteroides*	5.90	2.08	1.05	0.12

Fold change was estimated with age as a counfounder. P-value  was adjusted for multiple testing with Benjamini-Hochber method.

### Imputed Metagenomic Analysis Reveals Upregulation of Modules Related to Lipopolysaccharide Synthesis

**Figure 3:**
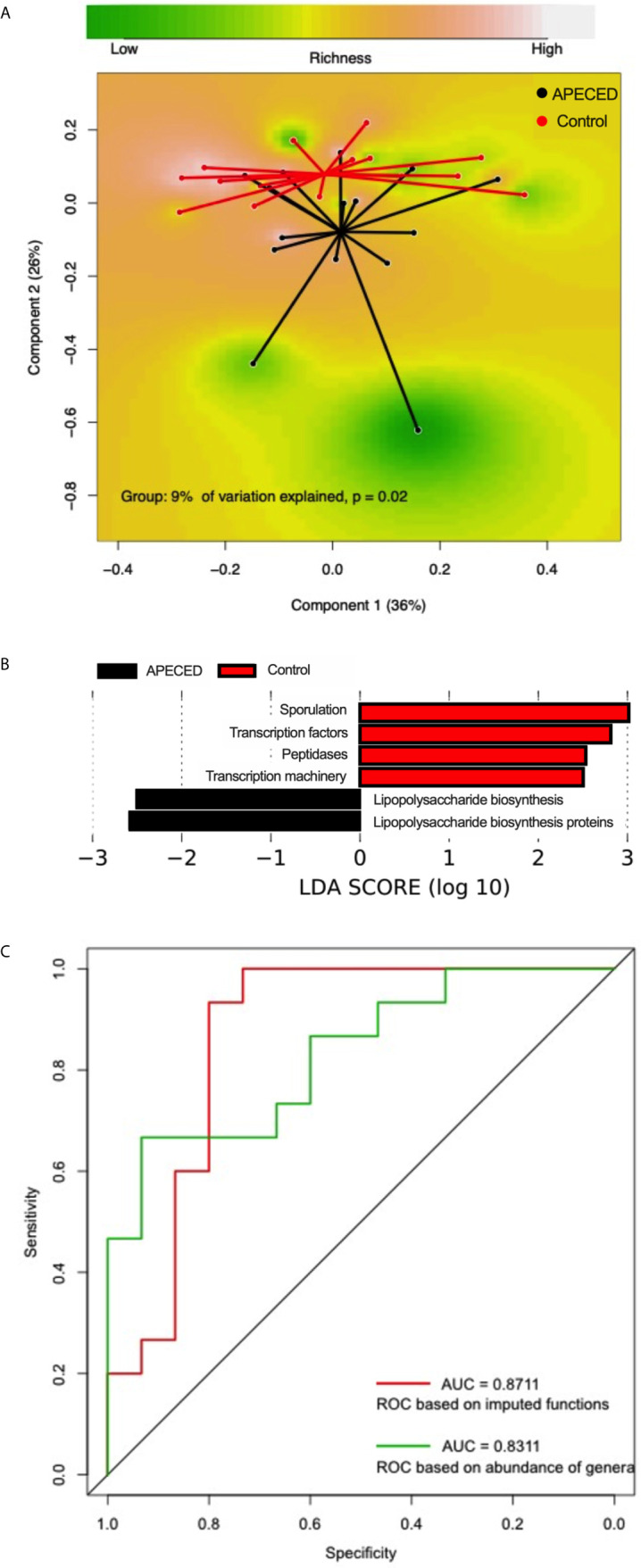
**(A)** Principal coordinate analysis (PCoA) based on Bray–Curtis distances using the predicted functional modules showing differences in the gut microbiota compositions between patients with APECED (black) and healthy controls (red). **(B)** Predicted functional modules significantly overrepresented in patients with APECED (black) and controls (red). **(C)** Receiver operating characteristic (ROC) curves of the cross-validated random forest models constructed using the imputed functions (red) and relative abundances of genera (green).

To understand functional implications of the observed taxonomic difference between patients with APECED and controls, we inferred metagenomes using the PICRUSt2 algorithm. This is a computational approach that reconstructs functional composition of a metagenome connecting the sequenced genes to reference genomes. The imputed functions of patients’ microbiota differed significantly from those of healthy controls, explaining 9% of the variation in the gut microbiota (*P* = 0.02, **Figure 3A**), which was attributable to the functional modules associated with LPS synthesis that were enriched in patients (**Figure 3B**).

Random forest (RF) classification models were subsequently trained by 5-fold cross-validation using the relative abundances of all genera (N = 65) or imputed functional modules (N = 211). Both RF models including bacterial taxa or predicted functions classified patients with APECED with a comparably robust accuracy, achieving an area under the curve (AUC) of 0.83 and 0.87, respectively (**Figure 3C**). The most important genera selected by the model included *Faecalibacterium* and *Atopobium* (**Supplementary Figure 1A**). The functional modules related to LPS synthesis were among the most important features selected by the random forest model (**Supplementary Figure 1B**).

### Dysbiosis in Patients With APECED Is Linked to ASCA Antibodies and Yeast Abundance in the Stool

**Figure 4:**
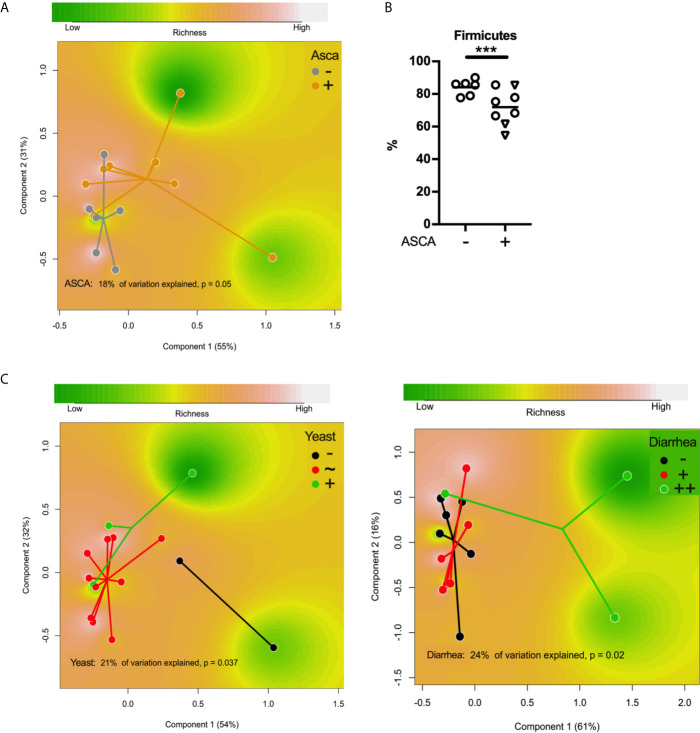
**(A)** Principal coordinate analysis (PCoA) based on Bray–Curtis distances using the phylum-level data showing differences in the gut microbiota compositions between ASCA-positive and ASCA-negative patients with APECED. **(B)** The relative abundance of Firmicutes in patients with or without ASCA. **(C)**Principal coordinate analysis (PCoA) based on Bray–Curtis distances using the genus-level data showing differences in the gut microbiota compositions between patients with APECED ranked according to abundance of yeast in stool and the severity of patient’s gastrointestinal symptoms, respectively. *** denotes p-value < 0.001; –, negative/low; +, positive; ++, markedly positive ie. severe symptoms; ~, average.

Having established the differences in the gut microbiota between patients and controls at the level of the entire bacterial community and individual taxa, we next examined whether patient characteristics were linked to gut microbiota deviations. We used the ASCA quantification that was available for all but one of the patients with APECED, and 8/14 (57.1%) were positive, indicating a systemic immune response to fungal products or fungi. Five of the ASCA positive patients had diarrhea including the three patients with most severe symptoms. The ASCA positivity explained 10% of variation in the gut microbiota on the phylum level (*P* = 0.05, **Figure 4A**). As mentioned previously, a lower proportion of Firmicutes was characteristic to patients compared with healthy controls, which was more pronounced in ASCA positive patients (FDR-*P* = 1.1x10^-6^ between ASCA+ and ASCA- patients, **Figure 4B**).

Interestingly, the antigen for ASCA antibodies can also be produced by *C. albicans* and infection by this pathogenic yeast has been shown to be able to induce ASCA (23). Patients with APECED are susceptible to chronic candidiasis (1, 2) and all of our patients had suffered from chronic candidiasis at some site of the body with varying severity. We used ITS1 primers to quantify fungi in patient stool microbiota by qPCR. Fungal DNA was amplified in all patient samples and based on the amplification results, the patients were grouped into three categories using a comparative Ct method (median Cq of patients +/- 1 cycles, respectively). Three of the patients had a markedly higher level of fungi in their stool, while two patients had a significantly lower level than other patients. Bacterial composition of these groups differed significantly (21% variation explained in PERMANOVA, *P* = 0.04, **Figure 4C**). Four patients were receiving anti-fungal medication at the time of sampling (one in the low group, the rest in the median group).

Autoimmunity against neuroendocrine cells as measured by TPH antibodies was detected in 60% of the patients (9/15) and was associated with a higher abundance of *Haemophilus* (FDR-*P* = 0.001, fc = 65 to patients without anti-TPH antibodies).

### Changes in Microbiota Are More Pronounced in Patients With Most Severe Gastrointestinal Symptoms

We examined the associations between the intestinal microbiota and GI symptoms of the patients. Four out of the fifteen patients (26.7%) suffered from intermitting obstipation episodes, but their stool microbiota did not differ from the other patients. The opposite was, however, true for patients reporting symptoms of diarrhea. We divided patients into three groups according to the severity of their symptoms of diarrhea (no symptoms, N = 6; intermittent diarrhea, N = 6; chronic difficult diarrhea, N = 3) and found the fecal microbiota composition to significantly differ among the groups (24% of variation explained in PERMANOVA on the family level, *P* = 0.02; 15% variation explained in PERMANOVA on the genus level, *P* = 0.02, **Figure 4C**). *Atopobium* spp. were overrepresented in patients with intermitted diarrhea and chronic difficult diarrhea (fc = 6.3, FDR-*P* = 2x10^-35^; fc = 117, FDR-*P* = 6x10^-305^, respectively, compared to patients without symptoms) while *Faecalibacterium* spp. were less common if a patient had chronic difficult diarrhea (fc = 0.3, FDR-*P* = 0.05 compared to patients without symptoms).

The microbial composition of patients without any GI symptoms (N = 5), also differed from the healthy controls (11% of variation explained in PERMANOVA on the genus level, *P* = 0.04).

## Discussion

Our gut microbiota analysis in patients with APECED revealed significant differences in the gut microbiota both at the level of the entire community and individual taxa compared to healthy controls. At the phylum level, we saw a reduction in gram-positive *Firmicutes* and increases in gram-negative *Bacteroidetes* and *Proteobacteria* in patients with APECED compared to the healthy controls. On the genus level, a reduction in the butyrate producing *Faecalibacterium* represented one of the most apparent features in the gut microbiota of patients with APECED. *Faecalibacterium prausnitzii* is known for its anti-inflammatory properties and it is shown to protect mice from effects of chemically induced colitis (24). Interestingly, a decrease in *Faecalibacterium* spp. has been consistently associated with disrupted gut homeostasis in inflammatory bowel disease (IBD) (9–11).

We detected *Atopobium* spp. in the stool samples from all patients with APECED, and in one patient it accounted up to 20.3% of bacteria. The relative abundance of *Atopobium* ssp. was especially high in patients with severe GI symptoms. *Atopobium* belongs to the Actinobacteria and is capable of producing H2S from sulphur containing amino acids and hence has been implicated in halitosis in the oral cavity (25). *Atopobium* is key to a network of H2S producing bacteria and correlates with the severity of Crohn’s disease (10). *Atopobium* has been linked to mitochondrial dysfunction, and the transfer of *Atopobium* triggers colitis in IL10-/- mice (10). Interestingly, *Atopobium* and *Megashaera* spp., enriched in the microbiota of patients with APECED compared to healthy controls, are known to grow in biofilms e.g. in the vaginal (26) and oral (27) ecosystems. Bacterial biofilms, mucosa-associated dense and resistant polymicrobial communities encased in extracellular matrix are very common in IBD patients and typically contain bacteria that can invade intestinal epithelia, potentiate pro-inflammatory signals and communicate *via* quorum sensing (28). It has been proposed that microbial biofilms are virtually absent in the gut of healthy subjects but their presence in patients’ intestine could represent an indicator of emerging disease (29). Also *Butyrivibrio* and *Prevotella*, enriched in the patients, may engage in biofilms based on their high genomic content for quorum sensing proteins (30).

In the study showing the pathogenic role of *Atopobium* in Crohn’s disease (10), the microbiome analysis was based on samples acquired by mucosal lavage during colonoscopy. It has been shown that samples obtained with endoscopy provide a more sensitive indication of differences between patients with GI disease and controls, whereas much of this difference is lost or blurred in stool samples (8). Thus, the significant increase of biofilm-producing bacteria in the stool samples from patients with APECED likely reflect a marked dysbiosis of the gut. Moreover, we found that the predicted function of LPS biosynthesis in the gut microbiota was significantly elevated in patients with APECED compared to controls, in line with the enrichment of several gram-negative bacterial genera. LPS is a pro-inflammatory molecule of the cell wall of gram-negative bacteria, which can contribute substantially to continued inflammatory response towards commensals, especially in an immunocompromised host and in the presence of disrupted barrier integrity.

A previous study using a smaller cohort of Finnish APECED patients identified an increase in *Haemophilus* genus in ten APECED patients compared to eight controls (5). Our data confirms this feature, but the differences in the gut microbiota between the patients and controls were more substantial and phylogenetically diverse in our study. The increase in strains of *Enterobacteriaceae* family belonging to either *Escherichia* or *Shigella* spp. reported in the previous study was not observed in our data, nor did we observe any differences in other genera belonging to the *Enterobacteriaceae* family. This difference is most likely explained by the fact that our study has a larger sample size, BMI-matched healthy controls from the same age range as the patients, and a fecal DNA extraction method that efficiently captures both gram-positive and gram-negative gut commensals (13). Moreover, two small studies on the salivary microbiota from six and seven APECED patients with age-matched controls found altered bacterial compositions in APECED patients’ oral cavity, but their results are mixed and in part discrepant (31, 32).

What, then, triggers the intestinal dysbiosis in APECED patients? Despite its monogenic background, the immunological phenotype of APECED is highly complex, and several factors may be linked to the alterations in intestinal microbiota. We have previously reported that patients with APECED have increased antibody responses against gut commensals (7). In the current study, ASCA antibodies were associated with decreased *Firmicutes* in patients with APECED. ASCA against oligomannose of the yeast were detected in Crohn’s disease where it was associated with more severe outcome and could be used as a serological marker of the disease (33, 34), but the clinical significance of these antibodies is unclear. The antibodies likely reflect the pathological process and are not pathogenic *per se*. Patients with APECED also have autoantibodies against Th17-cytokines, such as IL-22 and IL-17 (2). IL-22 contributes to the maintenance of barrier defense and integrity in the gut. Neutralizing antibodies against IL-17 have been linked to defective antifungal defense (2). Interestingly, in a small French study the GI symptoms of patients with APECED were alleviated with antifungal therapy, suggesting possible involvement of intestinal fungi (35). In Crohn’s disease, *C. albicans* colonization is more common compared to healthy controls and it is capable of initiating ASCA formation (23, 36). Indeed, *C. albicans* has been suggested to have a causative role in the initial GI inflammation (37). We found the abundance of yeasts in the fecal microbiota to be linked to bacterial alterations, but not to the severity of the GI symptoms experienced by the patients. However, as our samples were taken at the time of established disease, they may not reflect the situation during the initial disruption of gut homeostasis. Anti-cytokine antibodies have also been proposed to account for the bacterial alterations seen in APECED patients’ mouth (32).

Another hypothesis explaining the aberrant gut microbiota of patients with APECED is that autoimmunity against neuroendocrine cells or other components of the gut might lead to a disrupted intestinal barrier and subsequent pro-inflammatory intestinal conditions. *Haemophilus*, a well-known Gram-negative genus with members with pathogenic properties was associated with IBD in previous studies (8, 10). Of interest, the relative abundance of members of this genus appears to be very high in patients with anti-TPH antibodies. Finally, the failure of immunoregulatory mechanisms may also have a negative effect on gut homeostasis. Patients with APECED have a defect in regulatory T cells and we have previously shown that at least in some patients, regulatory T cells are decreased in the gut (7). Alterations in the adaptive immunity of patients can thus also contribute to the emergence of disturbances in the gut microbiota.

In summary, our data indicate that several gram-negative bacterial genera were enriched in the gut microbiota of patients with APECED, which may promote dysbiosis and the inflammatory phenotype *via* biofilm production and increased exposure to LPS. This microbiota profile in the patients was associated with more severe GI symptoms. Gastrointestinal manifestations of APECED were initially overlooked amidst severe autoimmune components of the disease, but a significant proportion of patients suffer from various disease manifestations of the GI tract, which can decrease the quality of life (3). Therefore, at least in patients with severe GI symptoms, the gut microbiota is a factor to take into consideration when contemplating therapy.

## Data Availability Statement

The datasets presented in this study can be found in online repositories. The names of the repository/repositories and accession number(s) can be found below: European Nucleotide Archive, accession no: PRJEB46143.

## Ethics Statement

The studies involving human participants were reviewed and approved by The Ethics committee of the Helsinki University Hospital. The patients/participants provided their written informed consent to participate in this study.

## Author Contributions

IH designed and performed experiments, analyzed data with CJ, and wrote the original draft of the manuscript with CJ and AS. SL and OM collected the patient samples and clinical data. A-MP collected the control samples. WV, TA, and AS designed experiments. All authors contributed to the article and approved the submitted version.

## Funding

This study was funded by the Emil Aaltonen Foundation, University of Helsinki, Academy of Finland (grant 308255), Sigrid Jusélius Foundation, Folkhälsan Research Foundation, Novo Nordisk Foundation, Helsinki University Hospital Research Funds, Pediatric Research Center, Helsinki University Hospital, The Finnish Foundation for Pediatric Research, and The Finnish Medical Foundation.

## Conflict of Interest

The authors declare that the research was conducted in the absence of any commercial or financial relationships that could be construed as a potential conflict of interest.
