# Supplementary figures and images for: Fecal Bacteria Implicated in Biofilm Production Are Enriched and Associate to Gastrointestinal Symptoms in Patients With APECED – A Pilot Study
Source: Front Immunol. 2021 Jul 22;12:668219. doi: 10.3389/fimmu.2021.668219 (PMC8339580; doi:10.3389/fimmu.2021.668219)

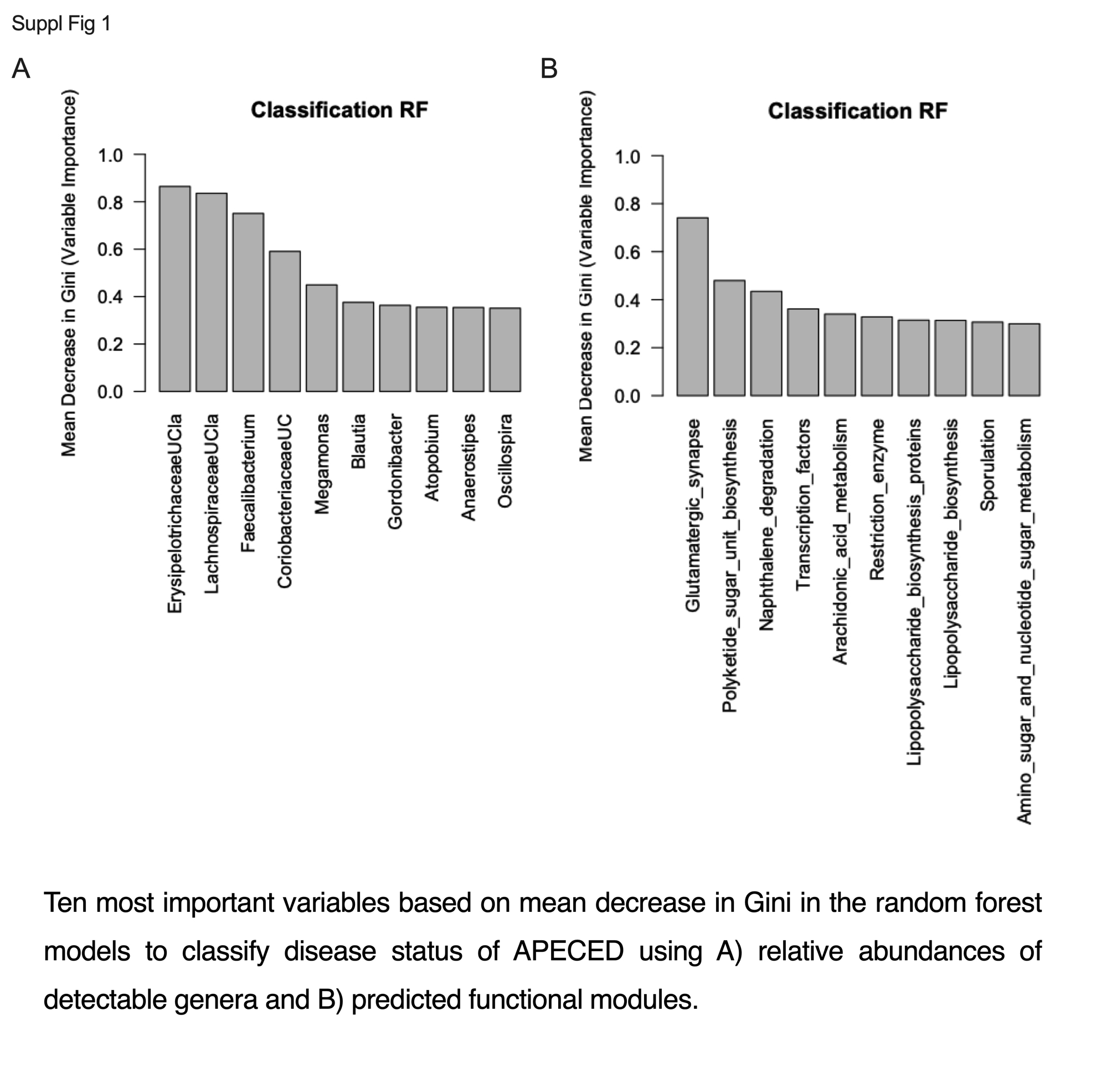

Supplement: Supplementary file 1 [file Image_1.tiff]
